# Supplementary material for: Associations between Subsequent Hospitalizations and Primary Ambulatory Services Utilization within the First Year after Acute Myocardial Infarction and Long-Term Mortality
Source: J Clin Med. 2020 Aug 5;9(8):2528. doi: 10.3390/jcm9082528 (PMC7464321; doi:10.3390/jcm9082528)
Supplement: Supplementary file 1 [file jcm-09-02528-s001.pdf]

Table S1. Adherence to the guideline-recommended medical therapy during the first year of the follow-up.

Parameter	Total	SLOS			p for trend	PAV		p
		0-1	2-7	≥8		<10	≥10	
n	8112	4474	1899	1793		2111	6001	
**Adherence, Mean (SD)**[1]								
Aspirin	75.95 (32.07)	77.81 (31.43)	75.73 (32.04)	71.39 (33.28)	<0.001	65.23 (36.89)	79.72 (29.28)	<0.001
Statins	66.20 (35.75)	68.27 (35.31)	67.01 (35.46)	60.01 (36.51)	<0.001	55.11(39.24)	70.11 (33.58)	<0.001
Beta blockers	61.37 (37.39)	60.81 (37.84)	63.10 (37.00)	60.94 (36.60)	0.906	52.10 (38.78)	64.64 (36.33)	<0.001
ACE/ARB	63.86 (37.84)	65.10 (38.11)	63.56 (37.84)	61.01 (36.98)	<0.001	54.06 (39.97)	67.31 (36.44)	<0.001
Total	66.85 (27.38)	68.00 (27.69)	67.34 (27.18)	63.34 (27.18)	<0.001	56.63 (31.66)	70.44 (24.72)	<0.001
**Adherence≥ 80%, n (%)**								
Aspirin	5118 (63.1)	2947 (65.9)	1188 (62.6)	983 (56.5)	<0.001	1069 (50.6)	4049 (67.5)	<0.001
Statins	4107 (50.6)	2348 (53.3)	977 (51.4)	746 (42.9)	<0.001	850 (40.3)	3257 (54.3)	<0.001
Beta blockers	3603 (44.4)	1974 (44.1)	876 (46.1)	753 (43.3)	0.863	735 (34.8)	2868 (47.8)	<0.001
ACE/ARB	3993 (49.2)	2302 (51.5)	935 (49.2)	756 (43.5)	<0.001	838 (39.7)	3155 (52.6)	<0.001
Total	1767 (21.8)	1067 (23.8)	406 (21.4)	294 (16.9)	<0.001	353 (16.7)	1414 (23.6)	<0.001

[1] The adherence was calculated as the rate of issued monthly prescriptions, throughout the first year following hospital discharge. Abbreviates: ACE, Angiotensin converting enzyme inhibitors; ARB, Angiotensin II receptor blockers.
